# Computer-aided engineering of stabilized fibroblast growth factor 21

**DOI:** 10.1016/j.csbj.2024.02.001

**Published:** 2024-02-07

**Authors:** Gabin de La Bourdonnaye, Tereza Ghazalova, Petr Fojtik, Katerina Kutalkova, David Bednar, Jiri Damborsky, Vladimir Rotrekl, Veronika Stepankova, Radka Chaloupkova

**Affiliations:** a Department of Experimental Biology, Faculty of Science, Masaryk University, Brno, Czech Republic; b Enantis Ltd., Biotechnology Incubator INBIT, Brno, Czech Republic; c Department of Biology, Faculty of Medicine, Masaryk University, Brno, Czech Republic; d Loschmidt Laboratories, Centre for Toxic Compounds in the Environment RECETOX, Faculty of Science, Masaryk University, Brno, Czech Republic; e International Clinical Research Center, St. Anne's University Hospital, Brno, Czech Republic

**Keywords:** Fibroblast growth factor 21, Protein engineering, Protein stabilization

## Abstract

FGF21 is an endocrine signaling protein belonging to the family of fibroblast growth factors (FGFs). It has emerged as a molecule of interest for treating various metabolic diseases due to its role in regulating glucogenesis and ketogenesis in the liver. However, FGF21 is prone to heat, proteolytic, and acid-mediated degradation, and its low molecular weight makes it susceptible to kidney clearance, significantly reducing its therapeutic potential. Protein engineering studies addressing these challenges have generally shown that increasing the thermostability of FGF21 led to improved pharmacokinetics. Here, we describe the computer-aided design and experimental characterization of FGF21 variants with enhanced melting temperature up to 15 °C, uncompromised efficacy at activation of MAPK/ERK signaling in Hep G2 cell culture, and ability to stimulate proliferation of Hep G2 and NIH 3T3 fibroblasts cells comparable with FGF21-WT. We propose that stabilizing the FGF21 molecule by rational design should be combined with other reported stabilization strategies to maximize the pharmaceutical potential of FGF21.

## Graphical Abstract

**Figure:**
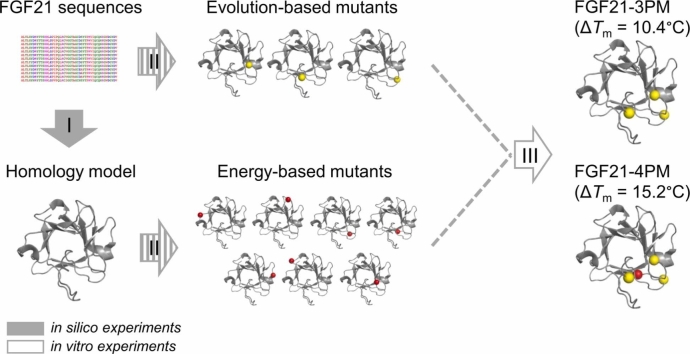

## 1 Introduction

The fibroblast growth factor (FGF) family is composed of signaling proteins secreted outside the cell (secreted FGFs) that activate tyrosine-kinase receptors and of intracellular non-signaling proteins (intracellular FGFs) that interact with voltage-gated sodium channels, and regulate the channel activity in neurons. Secreted FGFs regulate developmental pathways, playing a crucial role in the early embryo through the development of multiple organ systems. In adults, FGFs play the role of homeostatic factors controlling the maintenance and regeneration of tissues and playing an indirect role in metabolism. Most secreted FGFs (also called canonical FGFs) are responsible for signaling to nearby cells (paracrine signaling) and mostly diffuse through the extracellular matrix. FGFs bind to FGF receptors (FGFRs) with the help of a cofactor and kickstart a variety of signaling pathways, such as *MAPK/ERK*, *PI3K-AKT, STAT,* and *PLCγ*, that are involved in the control and regulation of cell proliferation, differentiation, and survival [1], [2], [3], [4], [5], [6]. However, three members of the secreted FGFs evolved to become endocrine signaling proteins that natively circulate the bloodstream and play a more diversified role. The endocrine FGFs control lipid, phosphate, and carbohydrate metabolism in addition to regular canonical FGF functions [1], [2], [7], [8], [9].

FGF21 is a member of the FGF19 subfamily and belongs to the endocrine FGFs. FGF21 was identified in 2000 by Nishimura et al. [10]. FGF21 was found to be predominantly expressed in the liver, and its functions were completely unknown until 2005 when it was presented as a novel metabolic regulator with potential for diabetes therapy [11]. The mature FGF21 protein, cleaved from its signal peptide (28 amino acids at the N-terminus), contains 181 amino acids and has an acidic theoretical isoelectric point of 5.4. Contrary to canonical FGFs, the cofactor mediating the interaction between FGF21 and FGFRs is a protein called β-Klotho [6], [12]. Studies on the tissue distribution of FGFR and β-Klotho determined that FGF21 primarily binds FGFR1c and FGFR3c [6], [13] although it can also bind FGFR2c and FGFR4 [14]. FGF21 lacks the high-affinity FGF-heparin binding domain, preventing its local immobilization in the extracellular matrix, thus allowing its secretion through the bloodstream. FGF21 up-regulates fatty acid β-oxidation, ketogenesis, and gluconeogenesis in the liver. It also stimulates insulin synthesis in pancreatic islets and white adipose tissue browning and glucose uptake [15]. These properties make FGF21 a molecule of interest for treating metabolic diseases such as obesity, dyslipidemia, or insulin intolerance [15]. However, FGF21's potential for therapeutic applications is severely limited by its intrinsic instability [16]. *In vivo*, the majority of FGF proteins, including FGF21, are prone to protease, heat, and acid-mediated degradation. Consequently, FGFs have a propensity to aggregate and precipitate in solution. This leads to rapid loss of their biological activity and short half-life *in vivo* and *in vitro* [17]. In addition to these issues, FGF21 is prone to being cleared by the kidneys due to its low molecular weight, leading to poor pharmacokinetics.

Increasing the thermal stability of FGFs is, therefore, crucial to unlocking their therapeutic potential [18], [19]. Most commonly, extrinsic strategies such as the design of excipients and delivery systems are used to ensure the delivery of FGFs to the therapeutic area and protection from degradation [20], [21], [22], [23], [24]. Additionally, a wide array of FGF proteins have been the subject of protein engineering strategies aiming to stabilize the protein intrinsically to enhance the protein stability, solubility, or production yield [25], [26], [27], [28], [29], [30], [31]. Due to the endocrine nature of FGF21 and its administration through the bloodstream, the engineering of FGF21 is facing additional challenges, such as avoiding early clearance by the kidneys or degradation by the many proteolytic pathways that can affect long-range signaling proteins. Kharitonenkov et al. provided the first proof-of-concept for rational engineering of stable FGF21 variants by introducing a disulfide bridge in the protein core and truncating four amino acids on the N-terminus [29]. Other groups proposed to solve the problem of low *in vivo* half-life by increasing the molecular weight of FGF21 since low molecular weight proteins are more prone to being cleared by the kidneys [32]. The preferred strategy for increasing protein molecular weight is site-specific PEGylation. An early PEGylated variant of FGF21 exhibited a longer-lasting activity *in vivo* compared to wild-type FGF21 despite a 6-fold decrease in potency. This resulted in equivalent treatment outcomes over the studied period [33], [34], thus confirming that improving the pharmacokinetics of FGF21 can compensate for lower intrinsic activity. Since then, new PEGylated FGF21 analogs with more conserved biological activity have been developped [35], [36]. Other high molecular weight FGF21 analogs such as Fc (crystallizable fragment domain of immunoglobulins) fused variants have also been reported [32], [37], [38]. However, even high molecular weight FGF21 analogs, protected from rapid kidney-mediated clearance, are still subject to multiple degradation pathways that are not well described such as the fibroblast activator protein meditated proteolysis [39], [40]. Combining Fc-fusion to two rationally designed substitutions, Hecht et al. constructed an FGF21 analog with improved pharmacokinetics and similar β-Klotho affinity when compared to native FGF21 [32].

FGF21 therapeutics are generally well tolerated in short-term animal and clinical trials [41]. Nevertheless, several side-effects such as changes in bone resorption markers reported for long-acting analogue of FGF21 formed by two modified FGF21 molecules linked to immunoglobulin 1 monoclonal antibody backbone [42] or increased synthesis of anti-FGF21 antibodies reported for PEGylated forms of FGF21 protein [43] were observed. A protein engineering approach based on intrinsic modification of amino acid sequence can potentially solve the issue of adverse effects likely caused by unnatural post-translational modifications of the protein chain or its covalent attachment to the larger molecule. The development of stable and long-acting FGF21 molecules can allow lower dosage and/or lower frequency of the protein administration, further reducing the side-effect intensity. In any case, for all recombinant or engineered FGF21 analogues, more research is needed to study potential long-term safety issues.

Interestingly, there is a distinct lack of reports of *in silico* energy-based and evolution-based approaches to design stabilizing mutations for FGF21 in the literature. These approaches search for mutations likely to stabilize a protein of interest *via* evaluation of the folding free energy change of all possible point mutants, referred to as “energy-based mutations”, and *via* phylogenetic analysis to identify residues that have drifted from more stable consensus sequence, referred to as “evolution-based mutations” [44], [45]. The computational approaches have been often applied for enzyme stabilization when the constructed thermostable single- and multiple-point variants simultaneously exhibited increased activity at higher temperatures [46], [47]. In the case of FGF protein stabilization, the computational design combining the energy-based and evolution-based calculations resulted in the protein variants where the enhanced melting temperature was positively correlated with prolonged bioactivity at 37 °C [26], maintained mitogenic activity upon preincubation at elevated temperatures, and enhanced resistance towards proteolytic degradation [48]. This motivates us to apply such computational design also for the stabilization of FGF21. The main obstacle hindering the application of computational design on stabilization of FGF21 was likely the scarcity of the structural data until recently [49]. Only two partial structures of FGF21 are available in the protein database: the crystal structure of the protein C-terminal part in complex with β-Klotho (PDB ID: 5VAQ) [12] and a very recent NMR structure of the protein core (PDB ID: 6M6E) [49]. At the same time, while the overall structure of FGFs is conserved, FGF21 is a unique case within the FGF family because it shares only 38% of sequence identity with its closest homologue, FGF19 [49].

In this article, we report the construction and characterization of thermostable and biologically active single-point and multiple-point mutants of FGF21 designed by computer-aided protein engineering strategy employing energy-based and evolution-based analyzes. The lack of structural data we overcome by building of FGF21 homology model, whose accuracy was assessed by comparison with available NMR structure. There were three main goals of the study. First, rationally design thermostable single- and multiple-point mutants of FGF21 by the protein engineering strategy that has not been previously applied for FGF21. Second, to explore how achieved improvement in protein thermostability affects the biological activity. Third, to identify whether the enhancement of structural stability (melting temperature) of the protein goes in line with the improvement of other stability parameters such as bioactivity preservation after preincubation at elevated temperature.

## 2 Material and methods

### 2.1 Construction of FGF21 homology model

Five homology models were constructed by servers Robetta and I-Tasser by fully automated mode, which selects a template for modeling. The structure of human FGF19 (PDB ID 2P23) [50] solved at resolution 1.8 Å was selected as a template. Obtained structural models of FGF21 were further evaluated using the web tool QMEAN [51], [52], [53], which compares energies of structural elements with similar proteins in the PDB database. Hydrogen atoms were then added to the model using H++ web server [54], [55], [56]. Because the used template had low sequence identity with FGF21, the structural models were refined using 20 ns Langevin molecular dynamics at 310 K using Amber 14 software [57]. Finally, the models were energetically minimized and clustered in the Amber 14 software [57]. A representative structural model was selected from the largest cluster. The accuracy of the predicted homology model of FGF21 was further assessed by its comparison with the NMR structure of FGF21 consisting of 10 conformers (PDB ID: 6M6E) [49]. RMSD values of all aligned Cα atoms between the homology model of FGF21 and all NMR conformers were calculated using the PyMOL [58].

The homology model of FGF21–4PM sequence was built using Swiss-Model [59] with default settings. The first conformer of the NMR of FGF21 structure (PDB ID: 6M6E) [49] was used as a template. Changes in side-chains interactions upon the introduction of the stabilizing mutations were analyzed by comparison of the homology model with the NMR structure of FGF21. The analysis was performed using the PyMOL [58].

### 2.2 Prediction of stabilizing effect of single-point mutations by energy-based approach

The constructed FGF21 homology model was used for the prediction of stabilizing mutation. The stability effects of all possible single-point mutations were estimated using the force-field calculations by FoldX [60] and Rosetta ddg monomer [61] programs. The threshold value for stabilizing mutation predicted by the energy-based approach was set to ΔΔG ≤ −1.0 kcal/mol. The threshold value for destabilizing mutation was set to ΔΔG > 1.0 kcal/mol. Mutations with the value of ΔΔG between these two thresholds were considered neutral. Conserved residues based on the ConSurf [62] calculation were discarded to preserve evolutionary important positions. Intramolecular ion interactions were calculated using the PIC server [63], and any mutations that would disrupt these interactions were excluded from further analyses. Functionally relevant positions, such as residues located in receptor- and β-Klotho-binding sites, were excluded from the selection to minimize mutations compromising protein function.

### 2.3 Prediction of stabilizing effect of single-point mutations by evolution-based approach

For the prediction of potentially stabilizing substitutions in FGF21 by evolution-based approach, multiple sequence alignment of FGF21 with related proteins was constructed. The amino acid sequence of human FGF21 (Supplementary Fig. S1) was used as a query for the PSI-BLAST [64] search against the NCBI non-redundant database. PSI-BLAST was performed with the E-value threshold of 10^−10^ for the initial BLAST search and a threshold of 10^−15^ for including the sequence in the position-specific matrix. Sequences collected after three iterations of PSI-BLAST were clustered by CD-HIT [65] at the 90% identity threshold. The resulting dataset of 1536 sequences was clustered with CLANS [66] using default parameters and varying P-value thresholds. Sequences clustered with FGF21 at the P-value of 10^−29^ were extracted and aligned with the MAFFT tool [67] incorporated in AliView alignment editor [68]. The alignment comprised 308 sequences from the FGF19 subfamily to which FGF21 belongs. For creating a representative and unbiased data set, UniqueProt [69] was used with the HSSP value set to 0.5. The final set of 58 sequences was used to estimate the level of conservation of individual sites within the FGF21-related proteins. Relative evolutionary rates for respective positions were calculated by ConSurf 2010 [62] using the empirical Bayesian method and JTT evolution model. The final alignment comprising 58 sequences was used as input for back-to-consensus analysis. The analysis was performed using the consensus cut-off of 0.5, which means that a given residue must be present at a given position in at least 50% of all analyzed sequences and not present in the wild-type sequence at the same time to be assigned as the consensus mutation. The final list of mutations was then filtered using two criteria: (i) ΔΔG value predicted by FoldX [60] does not exceed threshold 0.5 kcal/mol and (ii) selected residue does not interact by ion interactions with other residues within the protein structure.

### 2.4 Construction of FGF21 variants

The gene encoding the mature form of human FGF21 was optimized for expression in *Escherichia coli*, fused with N-terminal His-tag, commercially synthetized (GeneArt), and subcloned into the pET3c expression vector. Mutant recombinant genes were constructed using the NEBaseChanger v1.2.9 method and Q5 high-fidelity polymerase (New England Biolabs) according to the web program's instructions and the polymerase manufacturer's recommendations. Mutagenesis of FGF21 was done by whole plasmid PCR using the plasmid pET3c::His-*fgf21*, as a template and two reverse-complement oligonucleotides carrying desired mutations. The PCR products were transformed into NEB5α(DH5α) chemically competent *E. coli* cells. The resulting plasmids were isolated from overnight cultivation of DH5α cells using a GeneJet plasmid miniprep kit (Thermo Fischer Scientific). Multiple-point mutants (three-point mutant, FGF21–3PM; four-point mutant, FGF21–4PM; five-point mutant, FGF21–5PM) were constructed using the same protocol as single-point mutants. The following plasmids pET3c::His-*fgf21-Q104M*, pET3c::His-*fgf21–3PM*, and pET3c::His-*fgf21–4PM* were used as templates for the construction of FGF21–3PM, FGF21–4PM, and FGF21–5PM, respectively. The nucleotide sequences of the constructed FGF21 variants were verified commercially by the Sanger sequencing (LightRun, Eurofins Genomics).

### 2.5 Recombinant expression of FGF21 variants

Chemically competent *E. coli* Origami B(DE3) cells were transformed with the recombinant expression vectors (pET3c::His-*fgf21x*) using the heat shock method. The transformed cells were plated on LB agar plates supplemented with ampicillin (100 μg/ml) and incubated overnight at 37 °C. Single colonies were picked to be inoculated into 10 ml LB medium (Sigma–Aldrich) supplemented with ampicillin (100 μg/ml). The starter culture was grown overnight at 37 °C and used to inoculate 500 ml LB medium supplemented with ampicillin (100 μg/ml) in baffled 3 l-Erlenmeyer flasks. The cells were grown at 37 °C in a shaker running at 225 RPM. The overexpression of the FGF21 gene was induced when the optical density of the culture reached OD 0.6–0.8 at 600 nm, by the addition of isopropyl β-D-thiogalactopyranoside at a final concentration of 1 mM (Sigma–Aldrich). The cultivation was then incubated overnight at 20 °C with shaking at 225 RPM. The cells were harvested by centrifugation (4500 g, 4 °C, 20 min), washed in equilibrating buffer (20 mM KH_2_PO_4_, pH 7.5; 0.5 M NaCl, 10 mM imidazole) and resuspended in equilibrating buffer. This suspension was stored at − 80 °C.

### 2.6 Purification of FGF21 variants

The cell suspension was disrupted by sonication with the ultrasonic processor Hielscher UP200S (Hielscher) at 80% amplitude. The lysate was pelleted by centrifugation (21 000 g, 4 °C, 1 h). The cell-free extract (supernatant) was recovered. The recombinantly expressed FGF21 variants were purified from the cell-free extract using single-step Ni-NTA metallo-affinity chromatography. The cell-free extract was loaded onto a cOmplete His-Tag Purification Column (Merck) in equilibrating buffer (20 mM KH_2_PO_4_, pH 7.5; 500 mM NaCl, 10 mM imidazole). The same buffer was used to wash unbound and weakly bound proteins. The bound recombinant FGF21 variants were eluted by a linear gradient (0 – 100%) of elution buffer (20 mM KH_2_PO_4_, pH 7.5; 500 mM NaCl, 500 mM imidazole). Protein concentration was evaluated by the Bradford method [70]. The purity of the protein was checked by 10% sodium dodecyl sulfate polyacrylamide gel electrophoresis (SDS-PAGE) followed by Coomassie Brilliant Blue R-250 staining (Fluka). After purification, the protein samples were dialyzed overnight in 20 mM potassium phosphate buffer (pH 7.5) supplemented with 750 mM NaCl.

### 2.7 Determination of thermostability of FGF21 variants by differential scanning fluorimetry (DSF)

Freshly prepared FGF21 variants were concentrated to a final concentration of 2 mg/ml. The samples were filled into standard-grade capillaries (NanoTemper) and loaded into the Prometheus NT.48 nanoDSF (NanoTemper). The samples were continually heated from 20 to 90 °C at a temperature rate of 1 °C/min. The fluorescence signal excited at 295 nm with an excitation power of 60% was recorded at 330 and 350 nm. Unfolding transition points were determined from changes in fluorescence emission at 330 nm, 350 nm, and their ratios.

### 2.8 Determination of biological activity of FGF21 variants by activation of ERK pathway

The biological activity of FGF21 variants was assessed using the Hep G2 hepatocellular carcinoma cell line, which is commonly used as a liver *in vitro* model [71] and has been described to respond to FGF21 stimulation [72]. Hep G2 cell line was maintained in DMEM/F-12 1:1 (Thermo Fisher Scientific), supplemented with 10% fetal bovine serum, 2 mM L-glutamine, non-essential amino acids, and antibiotics penicillin and streptomycin in a humidified atmosphere with 5% CO_2_ at 37 °C. For the experimental procedure, cells were seeded into 12-well plates at 100 000 cells/well in 1 ml of cultivation media. While testing multiple-point mutants, cells were additionally starved in a serum-free medium for 24 h before the treatment to improve the signal-to-noise ratio. Cells were treated with FGF21 variants in a range of concentrations of 10, 50, 100, 500, and 1000 ng/ml. Cells treated with FGF1 were used as a positive control. FGF1 was used in a concentration 1000 ng/ml to ensure maximal ERK phosphorylation. Untreated cells were used as a negative control. Cells were treated for 5 and 15 min, after which they were harvested on ice (4 °C). Cells treated with single-point mutants were washed with PBS, lysed with 2x Laemmli buffer, and boiled at 95 °C for 3 min. Cells treated with multiple-point mutants were scraped in PBS, spun down (5000 g, 5 min), and lysed with 1% SDS lysis buffer (1% SDS, 50 mM Tris-HCl pH 6.8) containing 1x cOmplete EDTA-free protease inhibitor (Roche). Protein concentration in the lysate was determined using DC Protein Assay (Bio-Rad), adjusted to 1 mg/ml using the lysis buffer and 4x Laemmli buffer, and samples were boiled at 95 °C for 3 min.

Protein samples were resolved by SDS-PAGE (10% gels) and separated proteins were subsequently transferred to PVDF membranes (Merck Millipore). Membranes were then blocked in 5% dry milk in TBS-Tween for 1 h at room temperature and incubated overnight at 4 °C with primary antibodies. Anti-ERK rabbit antibody (CST #9102), anti-phospho-ERK rabbit antibody (CST #9101), anti-PCNA rabbit antibody (Sigma Aldrich HPA030522), anti-Lamin B1 goat antibody (Santa Cruz sc-6217), anti-α-tubulin mouse antibody (Exbio 11–250-M001), and anti-β-actin antibody (CST #3700) were used as a primary antibody in dilution 1:1000 in 5% dry milk in TBS-Tween. After a wash in TBS-Tween for 4 × 7 min, the membranes were incubated for 1 h at room temperature with horseradish peroxidase-conjugated anti-rabbit or anti-mouse secondary antibodies (Thermo Fisher Scientific). After the incubation, membranes were washed in TBS-Tween for 5 × 7 min and proteins of interest were visualized using Immobilon Western Chemiluminescent HRP Substrate (Merck Millipore) in the G:BOX detection system (Syngene). For the multiple-point mutant samples, membranes were stripped using Restore Western Blot Stripping Buffer (Thermo Fisher Scientific) for 20 min, washed in TBS-Tween, blocked in 5% dry milk in TBS-Tween for 1 h, and re-incubated with different primary antibody. Primary antibodies were used in the following order: anti-phospho-ERK, anti-ERK, and anti-β-actin. Signal density was measured as a mean grey value using Image Lab (Bio-Rad). In all experiments, pERK and ERK densities were normalized to loading controls from the same membranes, and the signal was expressed as a band density ratio of pERK/ERK relativized to the negative control.

Screening of biological activity of FGF21 single-point variants was performed in one biological replicate with a goal to quickly and qualitatively assess that introduced mutations do not compromise the protein's ability to activate the ERK pathway. Experiments comparing the biological activity of FGF21 multiple-point variants with FGF21-WT were conducted in 4 biological replicates. Bar graphs of the band density ratio of pERK/ERK, relativized to the negative control, for FGF21-WT and its multiple-point variants show mean ± standard error of the mean (SEM). Statistical analysis was performed by using GraphPad Prism software.

### 2.9 Determination of biological activity of FGF21 variants by cell proliferation assay

The activation of FGFR signaling results in a mitogenic response, which can be quantified using various cell proliferation/viability assays, *e.g.*, resazurin-based assay. Resazurin is a cell-permeable redox indicator and reflects cell viability by converting from a non-fluorescent blue dye resazurin to the highly fluorescent red dye resorufin in response to the chemical reduction of the growth medium resulting from cell growth. The fluorescent signal is proportional to the number of living cells in the sample. The biological activity of FGF21 variants was determined by a cell proliferation assay using Hep G2 and NIH 3T3 fibroblast cells. Hep G2 cells were seeded into a 96-well plate at 6000 cells/well in 150 µl cultivation media (DMEM, 10% FBS, 2 mM L-glutamine, 0.5 mM NEAA, Penicillin-Streptomycin). Three hours after seeding allowing proper cell adhesion, the cells were treated with the protein. FGF21 variants were serially diluted in cultivation media and 50 µl of protein solution was added to each well. Final concentrations of FGF21 variants ranged from 0.001 ng/ml to 2 µg/ml, each concentration was tested in triplicate. After four days of cultivation, 20 µl of resazurin solution was added to each well, and the plates were incubated overnight.

NIH 3T3 cells were seeded into a 96-well plate at 15000 cells/well in 150 µl assay media (DMEM, 0.5% NCS, Penicillin-Streptomycin, 2 µg/ml heparin, 2.5 µg/ml recombinant mouse β-Klotho). Three hours after seeding allowing proper cell adhesion, the cells were treated with the protein. FGF21 variants were serially diluted in assay media and 50 µl of protein solution was added to each well. Final concentrations of FGF21 variants ranged from 0.1 ng/ml to 1 µg/ml; each concentration was tested in triplicate. After 2 days of cultivation, 20 µl of resazurin solution was added to each well, and the plates were incubated for 8 h. Metabolic activity was measured by fluorescence reading (560 nm excitation, 590 nm emission) on Synergy 4H reader (BioTek Instruments).

Cellular proliferation caused by FGF21 is represented by the median effective dose, ED_50_ (concentration of protein needed to achieve half of the maximal proliferation) calculated using Quest Graph™ online ED_50_ calculator [73]. Data of resorufin fluorescence are presented as mean ± standard deviation (SD). Statistical analysis was performed by using GraphPad Prism software.

### 2.10 Thermostability determination of FGF21 variants by monitoring proliferation of Hep G2 cells

The ability of FGF21 variants to promote the proliferation of Hep G2 cells after exposure to 37 °C up to 10 days and 50 °C up to 3 days was tested to further explore the protein stability. Hep G2 cells were seeded into a 96-well plate at 7500 cells/well in 150 µl assay media (DMEM, 1% FBS, ZellShield). Protein treatment was administered to the cells 3 h after seeding to allow proper adhesion of cells. Prior to the testing, FGF21-WT, FGF21–3PM, and FGF21–4PM at the concentration 40 µg/ml were incubated at 37 °C and 50 °C for 1 day, 37 °C and 50 °C for 3 days and 37 °C for 10 days. After the preincubation, the protein solutions were serially diluted in cultivation media and 50 µl of protein solution in a final concentration ranging from 0.1 ng/ml to 10 µg/ml was added to the wells. Each protein concentration was tested in triplicate. Following 4 days of cultivation, 20 µl of resazurin solution was added to each well and the plates were incubated overnight. Measurement of metabolic activity was performed by fluorescence reading (560 nm excitation, 590 nm emission) on Synergy 4H reader (BioTek Instruments).

## 3 Results

### 3.1 In-silico design of stabilized single-point mutants of FGF21

To stabilize FGF21, we applied a computer-aided protein engineering approach combining *in-silico* and *in-vitro* steps. Initially, we constructed a homology model using FGF19 crystal structure (PDB ID 2P23) [50] as a structural template. FGF19 is the closest FGF21 homologue even though human FGF21 and FGF19 sequences share only 38% of sequence identity. To validate the accuracy of the homology model of FGF21 structure, we compared the predicted structural model with the recently solved NMR structure of FGF21 consisting of 10 conformers (PDB ID 6M6E) [49] in PyMOL [58]. The results of calculated RMSD values of all aligned Cα atoms (120 aligned atoms) between the homology model and the individual NMR conformers of FGF21 are summarized in Supplementary Table S1. The calculated RMSD values between the homology model and the individual NMR conformers lie in the same range as those of all aligned Cα atoms between individual FGF21 conformers (see Supplementary Table S2), implying the correctness of the predicted structural model of FGF21. The main difference between the homology model and NMR conformers of FGF21 was observed in the random coil formed by the amino acid residues 146–163, which is highly flexible in the NMR structure. It is therefore not surprising that the homology model does not align with the NMR structure well in this structural region (Supplementary Fig. S2). The other difference was identified in the small helix formed by the amino acid residues 53–56 which was not predicted in the homology model. The β-strands β4 and β5, which have the same conformation in all FGF21 conformers determined by NMR, are shifted in the homology model and the Cα atom of the proline residue located in the hairpin connecting these two sheets is shifted in the homology model by approximately 4 Å. The β-strands β1 and β12 are also slightly shifted in the homology model of FGF21. The rest of the homology model fits well with the NMR structure of FGF21.

Potentially stabilizing mutations in FGF21 were predicted using the FireProt method which combines evolution-based and energy-based approaches [44]. For the energy-based approach, changes in the free energy of all possible mutations were analyzed by Rosetta [74] and FoldX [60] based on previously described protocols according to Kellogg et al. [61] and Guerois et al. [75], respectively. ΔΔG free energies were collected over all 20 mutations in a particular position. Destabilizing mutations (ΔΔG > 1 kcal/mol), conserved residues, and residues important for the proper biological function of FGF21 were excluded from the selection to minimize the occurrence of mutations compromising protein function. The evolution-based approach uses a back-to-consensus analysis, which determines significantly conserved residues in related sequences based on a multiple sequence alignment. Identified residues were then filtered by FoldX [60] and Rosetta [74] using a threshold of free energy of ΔΔG < 0.5 kcal/mol.

**Fig. 1:**
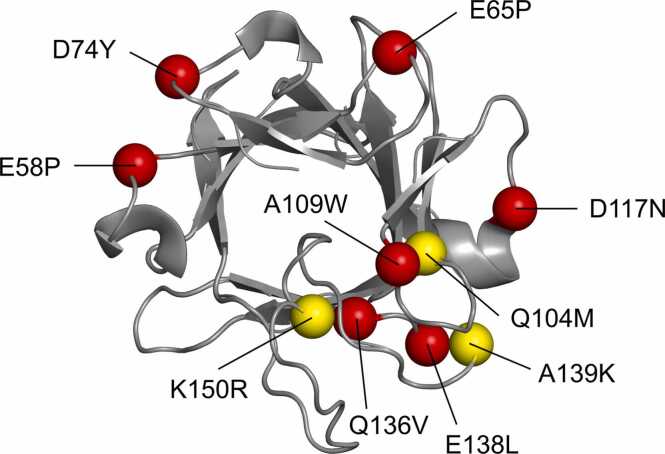
The structural model of FGF21 shown as a grey cartoon with visualized potentially stabilizing amino acid substitutions identified by energy-based (red spheres) and evolution-based (yellow spheres) predictions. The model of FGF21 structure was constructed by homology modeling using FGF19 crystal structure (PDB ID 2P23) [50] as a structural template.

A list of the best mutations identified by both energy-based and evolution-based approaches is summarized in Table 1, and their structural location is shown in Fig. 1. These single-point mutations were mostly located in structural regions with good alignment with the NMR structure. All identified mutations were selected for experimental validation.

**Table 1:** The stabilizing mutations identified in FGF21 by energy-based and evolution-based predictions. Mutations identified by the evolution-based approach are highlighted in bold.

Mutation*	ΔΔG^Rosetta^ (kcal/mol)	ΔΔG^FoldX^ (kcal/mol)	Conservation	Consensus	Amino acids in MSA
E58P	-3.2	-0.4	4	-	R,Q,D,L,N,Y,T,A,E,G,S,F,M,I
E65P	-3.0	-0.7	1	-	Y,T,S,A,G,E,H,M,R,K,D,L,P,N
D74Y	-2.4	-0.4	1	-	D,Q,V,Y,I,F,E,G,C,R,K,N,L,T,H,A,S
**Q104M**	-2.1	-1.8	9	0.81	Q,V,I,M,L
A109W	-1.9	1.1	5	-	K,Q,R,D,V,N,T,E,A,I,H
D117N	-1.9	0.2	6	-	E,S,M,I,H,T,L,V,N,K,Q,R,D
Q136V	-1.4	-0.3	2	-	T,Y,H,I,M,F,S,E,W,R,Q,K,V,L
E138L	-1.2	-1.2	3	-	E,A,S,M,T,L,P,N,Q,R,K,D,C
**A139K**	0.2	-0.6	6	0.50	R,Q,K,L,N,T,A,G,H
**K150R**	0.2	-0.4	4	0.52	K,R,Q,D,P,N,T,A,S,E,G,H

* The amino acid numbering corresponds to the sequence of human FGF21 (Supplementary Fig. S1). ΔΔG: predicted change in Gibs free energy; MSA: multiple sequence alignment.

### 3.2 Construction and biophysical characterization of FGF21 single-point mutants

The gene encoding humane mature FGF21 protein was commercially synthetized and subcloned in the pET3c vector, giving rise to the variant marked as FGF21-WT. All mutations herein are numbered according to the human FGF21 sequence, including the signal sequence (Supplementary Fig. S1). The ten identified stabilizing substitutions were constructed by site-directed mutagenesis. FGF21-WT and its ten single-point variants were recombinantly produced in *E. coli* in soluble form to a comparable yield of 6 mg of protein per liter of LB shake flask culture (with the experimental error of 10%). After purification of the protein samples, we tested the thermal unfolding of all FGF21 single-point mutants by DSF [76]. The experimental melting temperature (*T*_m_), defined as the temperature at which 50% of the protein sample is unfolded, is generally recognized as an accurate indicator of protein thermal stability [77], [78]. Eight out of ten constructed FGF21 mutants were found to be stabilizing. The mutants displaying the most significant increase in the melting temperature (FGF21-E58P, FGF21-A109W, FGF21-D117N, FGF21-Q136V, FGF21-Q104M, FGF21-A139K, FGF21-K150R) compared to FGF21-WT (Δ*T*_m_) were selected for further biological activity testing (Table 2).

**Table 2:** Melting temperature of FGF21-WT and its single-point mutants determined by DSF. The mutants exhibiting significant improvement in melting temperature compared to FGF21-WT (highlighted in bold) were selected for biological activity testing.

Variant	*T*_m_ (°C)	Δ*T*_m_ (°C)
FGF21-WT	44.4 ± 0.05	-
**FGF21-E58P**	47.3 ± 0.12	2.9
FGF21-E65P	44.6 ± 0.05	0.2
FGF21-D74Y	45.6 ± 0.17	1.2
**FGF21-A109W**	47.7 ± 0.24	3.3
**FGF21-D117N**	50.3 ± 0.11	5.9
**FGF21-Q136V**	53.4 ± 0.36	9.0
FGF21-E138L	43.3 ± 0.08	-1.1
**FGF21-Q104M**	51.0 ± 0.16	6.6
**FGF21-A139K**	49.0 ± 0.12	4.6
**FGF21-K150R**	48.4 ± 0.27	4.0

*T*_m_: melting temperature; Δ*T*_m_: change in melting temperature due to introduced mutation.

### 3.3 Screening of the single-point mutant biological activity

Screening of biological activity of FGF21 single-point variants was performed in one biological replicate with a goal to quickly and qualitatively assess that introduced stabilizing mutations do not destroy the protein's ability to activate the ERK pathway. As a model cell line for *in vitro* testing of FGF21 mutants’ biological activity, we used the Hep G2 hepatocellular carcinoma cell line. This cell line is commonly used as a liver model for *in vitro* testing and is responsive to FGF21 stimulation [72]. Upon binding of FGF21, FGFR1 activates the mitogen-activated protein kinase (MAPK) pathway, which regulates its downstream targets involved in metabolic regulation like glucose transporter 1 [11]. In the MAPK pathway, the signal is conveyed by a cascade of phosphorylation reactions. The biological activity of FGF21 mutants was measured as the amount of phosphorylated (active) extracellular signal-regulated kinase (ERK), the last part of the MAPK pathway, using western blot with antibodies specific to the total and phosphorylated form of ERK. The activity screening of the seven FGF21 single-point variants was divided into three experiments. In each experiment, the ERK activation induced by a particular mutant was qualitatively compared with that of the FGF21-WT. Due to the relatively high scatter of pERK signal observed for FGF21-WT in the individual experiments, we only qualitatively assess (yes/no assessment) the ability of the variants to induce ERK activation after 5 and 15 min of treatment. Although the quality of the blots is limited, observed data confirm that all tested single-point mutants are biologically active (Supplementary Fig. S3 and Fig. S4).

### 3.4 Construction and biophysical characterization of FGF21 cumulative mutants

The experimentally confirmed stabilizing mutations with uncompromised biological activity were combined into three cumulative FGF21 mutants. At first, we combined the three stabilizing substitutions that were predicted by the evolution-based approach. The constructed three-point mutant (FGF21–3PM) contains the following substitutions: A139K, K150R, and Q104M. Then, we combined FGF21–3PM with the most stabilizing substitution Q136V predicted by the energy-based approach leading to the construction of four-point mutant (FGF21–4PM) carrying the substitutions A139K, K150R, Q104M, and Q136V. Finally, we constructed five-point mutant (FGF21–5PM) that contains the substitutions A139K, K150R, Q104M, Q136V, and A109W. The melting temperature of each individual cumulative mutant was evaluated by DSF. The determined melting temperature of FGF21–3PM (54.8 °C) represents a 10.4 °C improvement compared to FGF21-WT. The *T*_m_ of FGF21–3PM was about 4.8 °C lower than the theoretical sum of contributions of the three individual substitutions, suggesting that the mutations have no strikingly additive stabilizing effect. FGF21–4PM carrying the additional substitution Q136V exhibited an additional 4.8 °C improvement in experimental melting temperature when compared to FGF21–3PM. The determined improvement in *T*_m_ of FGF21–4PM was again lower than the expected contribution of the Q136V substitution. The addition of A109W substitution into the FGF21–5PM even caused a 1.5 °C drop in the protein stability when compared to FGF21–4PM (Table 3) implying a synergistic effect of this mutation. Therefore, we selected FGF21–3PM and FGF21–4PM for detailed *in vitro* activity testing.

**Table 3:** Melting temperature of FGF21-WT and its cumulative multiple-point mutants determined by DSF.

Variant	*T*_m_ (°C)	Δ*T*_m_ (°C)
FGF21-WT	44.4 ± 0.05	-
FGF21-3PM (Q104M+A139K+K150R)	54.8 ± 0.12	10.4
FGF21-4PM (Q104M+A139K+K150R+Q136V)	59.6 ± 0.26	15.2
FGF21-5PM (Q104M+A139K+K150R+Q136V+A109W)	58.1 ± 0.08	14.1

*T*_m_: melting temperature; Δ*T*_m_: change in melting temperature due to introduced mutations.

### 3.5 Biological activity testing of FGF21 cumulative mutants

**Fig. 2:**
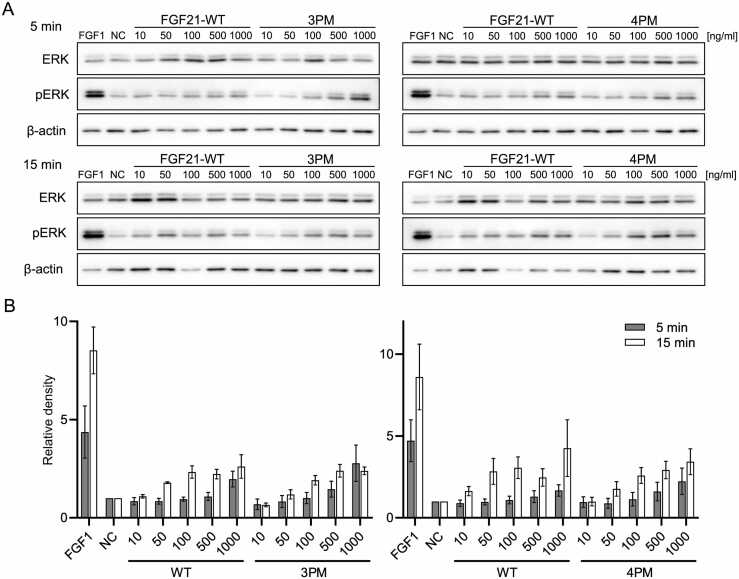
Determination of biological activity of FGF21 cumulative mutants by activation of ERK pathway. (A) Representative western blots of total ERK and phosphorylated ERK (pERK) in Hep G2 cells treated with FGF21-WT, FGF21–3PM, and FGF21–4PM in different concentrations after 5 and 15 min of treatment. 1000 ng/ml FGF1 was used as a positive control; untreated cells were used as a negative control; β-actin was used as a loading control. (B) Densitometric analysis of Western blots. The bar plots show the band density ratio of pERK/ERK, relativized to the negative control. The bars represent the mean ± SEM, n = 4. P value over the significance threshold (Wilcoxon signed-rank test).

The biological activity of selected cumulative mutants was tested by monitoring their ability to activate ERK phosphorylation in Hep G2 cells. FGF21–3PM and FGF21–4PM exhibited dose-dependent ERK phosphorylation in Hep G2 cells after 5 and 15 min similar to ERK phosphorylation observed with FGF21-WT (Fig. 2). This indicates that combining stabilizing single-point mutations did not negatively affect FGF21's ability to bind to FGFR and initiate the MAPK/ERK pathway.

**Fig. 3:**
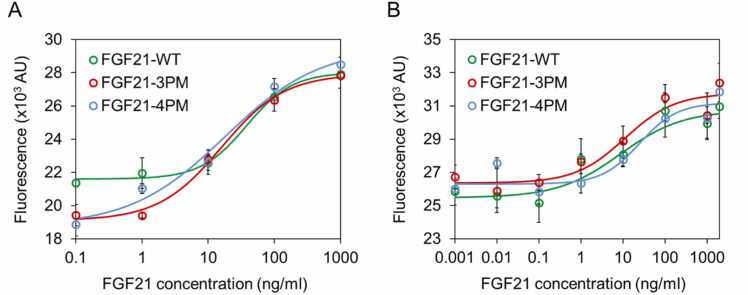
Biological activity of FGF21 variants measured in a cell proliferation assay using (A) NIH 3T3 fibroblasts in the presence of mouse β-Klotho and (B) Hep G2 cells. ED_50_ values for FGF21-WT, FGF21–3PM, and FGF21–4PM, determined in cell proliferation assay using NIH 3T3 fibroblasts, were 38.0, 15.9, and 18.0 ng/ml, respectively. ED_50_ values for FGF21-WT, FGF21–3PM, and FGF21–4PM, determined in cell proliferation assay using Hep G2 cells, were 8.7, 11.3, and 24.9 ng/ml, respectively. Data are presented as mean ± SD, n = 3. P value over the significance threshold (one-way ANOVA, Tukey’s multiple comparisons test). Solid lines represent the fit of the sigmoidal model to the experimental data.

The biological activity of FGF21-WT, FGF21–3PM, and FGF21–4PM was further determined by cell proliferation assay using Hep G2 hepatocellular carcinoma cells and NIH 3T3 fibroblasts. FGF21-WT and multiple-point mutants effectively induced the proliferation of Hep G2 and NIH 3T3 cells (Fig. 3). A comparison of the median effective dose (ED_50_) revealed that both thermostable multiple-point mutants are similarly effective in inducing the proliferation of both tested cell lines. Both multiple-point mutants also exhibited very similar maximal growth responses (*i.e*. same amplitude of the fluorescence signal change) when applied to Hep G2 cells. In the case of NIH 3T3 cells, the apparent higher maximal growth response observed for both multiple-point mutants when compared with FGF21-WT is likely attributed to the outlier point at the lowest dose of FGF21-WT rather than the changes in the protein efficiency to induce the cell proliferation.

The ability of FGF21 variants to promote cell proliferation after the exposure to elevated temperature was tested to further explore their stability. FGF21-WT, FGF21–3PM, and FGF21–4PM were exposed to 37 °C for 1–10 days and 50 °C for 1–3 days and then their ability to induce proliferation was determined in the Hep G2 cells. No difference between the individual protein’s residual bioactivity was identified (Supplementary Fig. S5). Preincubation of all three FGF21 variants at 37 and 50 °C for 1 day did not significantly affect the protein ability to effectively induce cell proliferation. With prolonged time of incubation at 37 °C, the activity of all three proteins gradually decreased in the same manner. Interestingly, upon 10 days of incubation at 37 °C, all FGF21 variants still exhibited residual ability to promote proliferation of Hep G2 cells, while 3 days of incubation at 50 °C led to complete loss of their activity.

### 3.6 Analysis of the structural base of the contribution of individual substitution to FGF21-4PM stability

In order to get structural insight into the protein stabilization, the effect of individual substitutions of the stabilized cumulative mutants was investigated. For this purpose, we constructed a homology model of FGF21–4PM using the first conformer of FGF21 NMR structure (PDB ID 6M6E) [49] as a template. The predicted homology model of FGF21–4PM was compared with the NMR structure of FGF21 and the changes in structure and side-chain interactions upon the introduction of the stabilizing mutations were analyzed. The introduction of the Q104M mutation caused the loss of one hydrogen bond between the side chain of Q104 and the backbone of P143 but probably led to better space filling of the protein core caused by the bulky side chain of Met residue. This is consistent with the computational design because although the Q104M is an evolution-based mutation, it was also ranked among the best stabilizing mutations by energy-based calculations. The A139K mutation led to the formation of a new hydrogen bond between the side chain of K139 and the backbone of M194. The effect of K150R mutation is difficult to explain because the mutation is located in one of the most flexible regions of the protein (loop formed by the residues 146–163) and also represents a type of conservative substitution. The Q136V mutation caused the loss of the hydrogen bond formed by the side chain of Q136 and the backbone of P143. Although Q136V was predicted by the energy-based approach and experimentally confirmed as the most stabilizing out of the tested mutations, the structural base of its contribution to the protein stability is difficult to explain.

## 4 Discussion

Here, we describe the use of rational design combining evolution-based and energy-based approaches that lead to the construction of single-point and multiple-point mutants of FGF21 with increased melting temperature *in vitro* and uncompromised biological activity. We demonstrate that stabilized multiple-point variants FGF21–3PM and FGF21–4PM are fully biologically active, as demonstrated by their ability to activate the ERK signaling pathway in Hep G2 cell culture and stimulate the proliferation of Hep G2 and NIH 3T3 fibroblasts cells with the same efficacy as FGF21-WT. Tsukada et al. investigated the correlation between the proliferation response of Hep G2 cells to hepatocyte growth factor (HGF) and the level of ERK activity induced by HGF [79]. Their results suggest that the weak activation of ERK induces the proliferation stimulation of Hep G2 cells, whereas the strong activation of ERK induces the proliferation inhibition. In our experiments, the level of ERK activation induced by both stabilized multiple-point mutants and the wild-type correlates with the observed proliferation stimulation induced by all three FGF21 variants.

We further investigate the residual activity of cumulative mutants FGF21–3PM and FGF21–4PM in cell proliferation assay following the preincubation at 37 and 50 °C for up to 10 and up to 3 days, respectively. Observed differences in background fluorescence between the individual experiments can be attributed to several factors associated with cell cultures, such as passage number, cell batch, confluency at cell harvesting, and slight variations in incubation times after addition of resazurin (especially in case of overnight incubation). We found out that enhanced structural stability of FGF21 variants does not correlate with the improved ability to maintain biological activity after exposure to elevated temperature. No difference between the residual proliferation activity of the mutants and FGF21-WT was identified. All protein variants maintain the original ability to promote the proliferation of Hep G2 cells after one day of incubation at both 37 and 50 °C. With a prolonged period of incubation at elevated temperatures, the bioactivity of all variants identically gradually decreased. In a recent study, Jung et al. discovered the temperature-responsive structural reversibility of FGF21-WT [80]. The authors found out that the heating of FGF21-WT solution up to 100 °C for 10 min immediately followed by a cooling down to 20 °C does not affect the protein structure and function. The described reversibility of the thermal unfolding of FGF21-WT could explain our observation that enhanced structural stability does not correlate with the simultaneously improved residual proliferation activity of FGF21 variants. Nevertheless, long-term exposure to high temperature still leads to loss of FGF21 biological activity as FGF21-WT and its stabilized variants did not induce proliferation of Hep G2 cells after 3 days of incubation at 50 °C.

We attempted to elucidate the structural base of the contribution of the four stabilizing substitutions introduced into both FGF21 multiple-point mutants. The introduced substitutions were localized in the different regions of FGF21 structure except for the hydrophobic core and biologically relevant interfaces that were omitted from the initial design. Both FGF21–3PM and FGF21–4PM carry three evolution-based mutations (Q104M, A139K, and K150R) that are further combined with energy-based mutation (Q136V) in the case of FGF21–4PM variant. A comparison of the homology model of FGF21–4 PM with the NMR structure of FGF21-WT did not reveal any explanation of the protein stabilization. The identified loss of one hydrogen bond compensated by the better filling of the protein core accompanied by the formation of one new hydrogen bond can hardly explain the improvement in melting temperature by 15 °C. The majority of the introduced stabilizing substitutions were predicted by evolution-based approach which identifies conserved residues in multiple sequence alignment of related sequences that are not present in the target protein [44]. Evolution-based mutations thus can be difficult to explain because natural selection can be affected by diverse parameters such as stability and biological function, accessibility of native states and folding pathways [81]. In our previous work aimed at understanding the molecular basis of the evolution-based mutation in haloalkane dehalogenase enzyme, we found out that the stabilization of the mutations identified by the evolution-based approach is driven by both entropy and enthalpy contribution, contrary to primarily enthalpy-driven energy-based mutations [45]. Stabilization of evolution-based mutations thus can be explained by differences in the solvation, flexibility, and energy of the unfolded state upon introduced mutation. These properties would be difficult to detect by structural analysis even if the crystal structure of the FGF21–4PM mutant was available.

The low intrinsic stability and limited blood retention are major issues for the potential use of FGF21 as therapeutics in metabolic diseases such as diabetes. Efforts to improve FGF21 stability and extend blood retention have included rational design, protein fusion, and covalent modification (PEGylations) [29], [30], [32], [33], [49]. Following the recent solving of the NMR structure of the FGF21 protein core, Zhu et al. achieved significant improvement of FGF21 by introducing a disulfide bridge to stabilize its β2-β3 region, which was shifting between an ordered hairpin-like structure and a highly disordered structure [49] while PEGylation and protein fusion strategies have shown success in increasing FGF21 blood retention [32], [33]. The goal of our study was to broaden the spectrum of methodology used to increase the FGF21 stability. We propose that the *in-silico* workflow used to successfully predict experimentally verified stabilized FGF21 mutants with uncompromised biological activity can be used in combination with other reported strategies to improve the potential of FGF21 as metabolic disease therapeutics.

## 5 Conclusion

By using a rational *in silico* design strategy combining evolution-based and energy-based approaches, we generate two multiple-point mutants of FGF21 with significantly enhanced melting temperature. Both multiple-point FGF21 mutants exhibited similar ability to induce ERK signaling in Hep G2 cells and comparable proliferation activity to that of FGF21-WT in Hep G2 and NIH 3T3 cell cultures. Additionally, we investigated the proliferation stimulation of Hep G2 cells by stabilized FGF21 variants preincubated at elevated temperatures. We found out that enhanced structural stability of FGF21 variants does not correlate with the improved ability to maintain biological activity after exposure to elevated temperature. The stabilized FGF21 variants exhibited comparable proliferation activity to FGF21-WT after the preincubation at 37 °C for up to 10 days and after the preincubation at 50 °C for 1 day. Our results demonstrate that an applied protein engineering strategy combining evolution-based and energy-based predictions of stabilizing mutations simultaneously conserving the biological activity can be used for the improvement of FGF21 properties. Finally, we propose that our protein engineering approach can be further combined with other reported stabilization strategies to maximize the pharmaceutical potential of FGF21.

## CRediT authorship contribution statement

V.S. and R.C. conceived the project; J.D., V.R., and R.C. supervised the project; T.G. and D.B. performed homology modeling and *in silico* design of protein variants; G.B. constructed protein variants; G.B., P.F., and K.K. carried out the functional characterization of proteins; G.B. and R.C. wrote the manuscript and prepared the figures; all authors have read and approved the manuscript.

## Declaration of Competing Interest

Jiri Damborsky, Veronika Stepankova, and Radka Chaloupkova are shareholders of Enantis Ltd, which is the first biotechnology spin-off company of Masaryk University. Gabin de La Bourdonnaye, Tereza Ghazalova, and Katerina Kutalkova are employees of Enantis Ltd. The authors Petr Fojtik, David Bednar, and Vladimir Rotrekl have no conflicts of interest with the contents of this article.
