# Supplementary material for: Computer-aided engineering of stabilized fibroblast growth factor 21
Source: Comput Struct Biotechnol J. 2024 Feb 7;23:942–51. doi: 10.1016/j.csbj.2024.02.001 (PMC10877085; doi:10.1016/j.csbj.2024.02.001)
Supplement: Supplementary file 1 — Supplementary material [file mmc1.docx]

Computer-aided engineering of stabilized fibroblast growth factor 21

Gabin de La Bourdonnaye^1,2^, Tereza Ghazalova^2^, Petr Fojtik^3^, Katerina Kutalkova^2^, David Bednar^4,5^, Jiri Damborsky^4,5^, Vladimir Rotrekl^3,5^, Veronika Stepankova^2^, Radka Chaloupkova^1,2,*^

^1^Department of Experimental Biology, Faculty of Science, Masaryk University, Brno, Czech Republic

^2^Enantis Ltd., Biotechnology Incubator INBIT, Brno, Czech Republic

^3^Department of Biology, Faculty of Medicine, Masaryk University, Brno, Czech Republic

^4^Loschmidt Laboratories, Centre for Toxic Compounds in the Environment RECETOX, Faculty of Science, Masaryk University, Brno, Czech Republic

^5^International Clinical Research Center, St. Anne's University Hospital, Brno, Czech Republic

*Corresponding author: Radka Chaloupkova, Biotechnology Incubator INBIT, Kamenice 34, 62500 Brno, Czech Republic, email: chaloupkova@enantis.com, phone: +420511205286

Table S1. RMSD values for the overlaps of FGF21 homology model’s Cα atoms with those of individual conformers of FGF21 structure solved by NMR (PDB ID 6M6E) [1].

| **FGF21 homology model  overlapped with conformer number** | **RMSD (Å)** |
| --- | --- |
| 1 | 2.75 |
| 2 | 2.57 |
| 3 | 2.77 |
| 4 | 2.99 |
| 5 | 3.14 |
| 6 | 2.54 |
| 7 | 2.80 |
| 8 | 2.70 |
| 9 | 2.98 |
| 10 | 2.39 |

Table S2. Comparison of individual conformers of FGF21 structure solved by NMR (PDB ID 6M6E) [1].

| **RMSD values for the overlaps of individual FGF21 conformer’s Cα atoms (Å)** | | | | | | | | | | |
| --- | --- | --- | --- | --- | --- | --- | --- | --- | --- | --- |
| **#** | **1** | **2** | **3** | **4** | **5** | **6** | **7** | **8** | **9** | **10** |
| **1** | - | 1.70 | 1.92 | 2.07 | 1.31 | 1.62 | 1.89 | 1.75 | 2.41 | 1.51 |
| **2** |  | - | 1.21 | 2.79 | 1.92 | 1.69 | 2.08 | 1.47 | 1.96 | 1.55 |
| **3** |  |  | - | 2.65 | 1.82 | 1.52 | 2.20 | 1.52 | 1.75 | 1.37 |
| **4** |  |  |  | - | 1.86 | 1.79 | 1.77 | 2.55 | 2.95 | 1.88 |
| **5** |  |  |  |  | - | 1.53 | 1.88 | 1.80 | 2.34 | 1.74 |
| **6** |  |  |  |  |  | - | 1.87 | 1.75 | 1.73 | 1.04 |
| **7** |  |  |  |  |  |  | - | 2.16 | 2.80 | 1.88 |
| **8** |  |  |  |  |  |  |  | - | 2.14 | 1.77 |
| **9** |  |  |  |  |  |  |  |  | - | 1.89 |
| **10** |  |  |  |  |  |  |  |  |  | - |


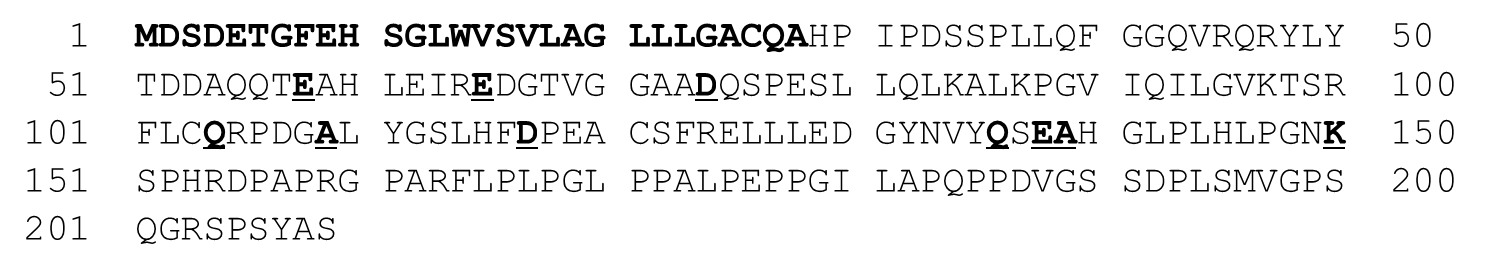


Figure S1. Amino acid sequence of human FGF21. The signal sequence is marked in bold, the amino acids mutated in this study are marked in bold and underlined.


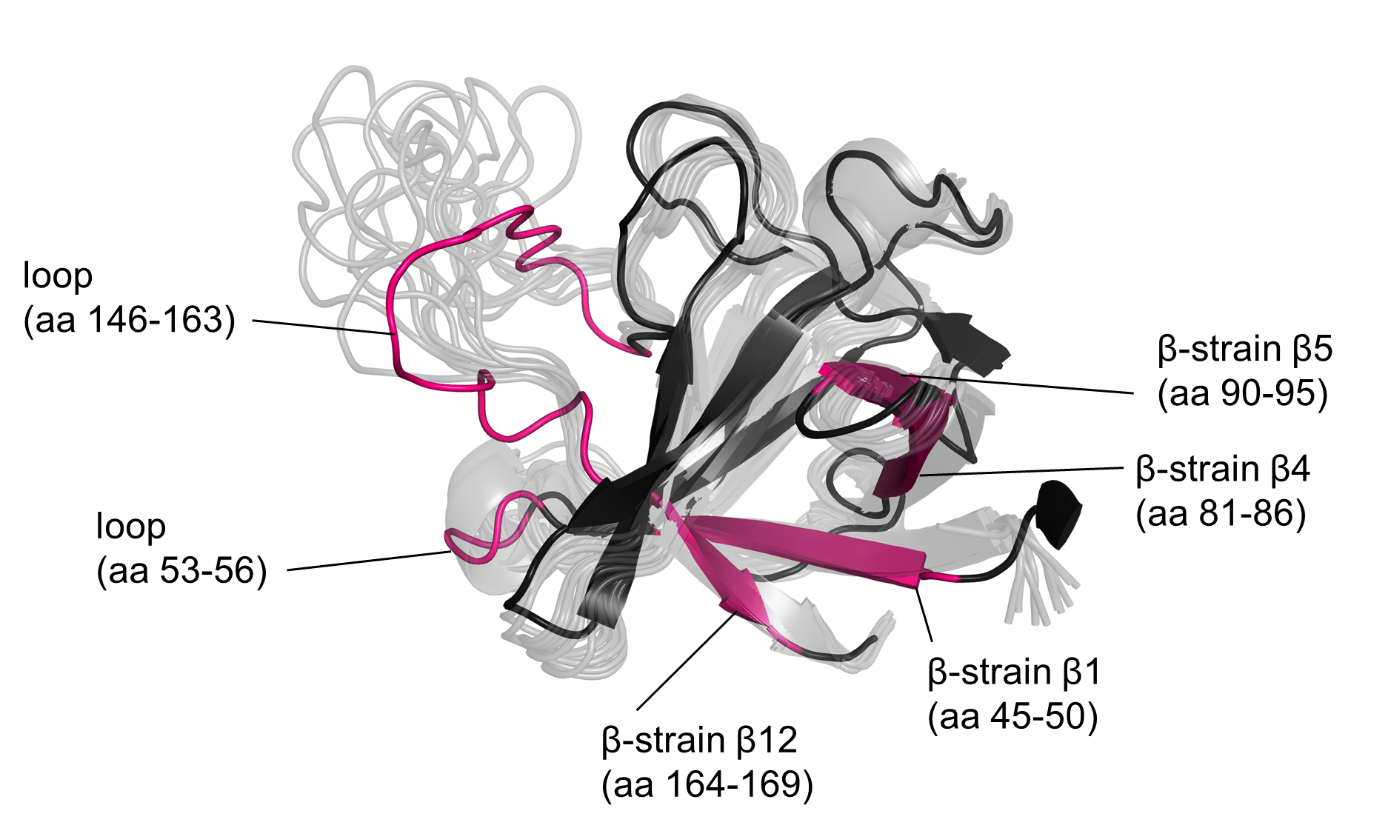


Figure S2. Superimposition of FGF21 homology model and NMR structure of FGF21 (PDB ID 6M6E) [1] consisting of 10 conformers. The model of FGF21 structure was constructed by homology modelling using FGF19 crystal structure (PDB ID 2P23) [2] as a structural template. Homology model of FGF21 is shown as black cartoon, the individual conformers of FGF21 structure are shown in grey. The main differences between the homology model and individual conformers of NMR structure of FGF21 are highlighted by pink color.


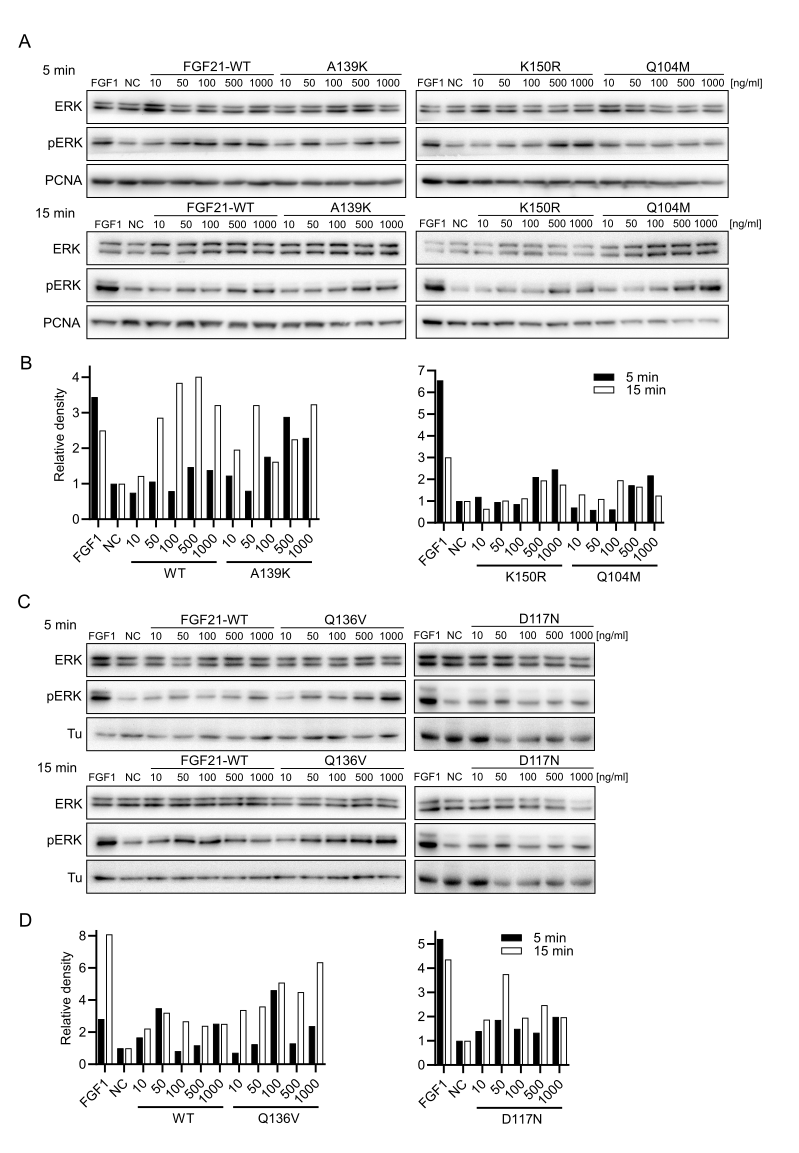


Figure S3. Determination of biological activity of FGF21 variants by activation of ERK pathway. (A) Western blots of total ERK and phosphorylated ERK (pERK) in Hep G2 cells treated with FGF21-WT, FGF21-A139K, FGF21-K150R, and FGF21-Q104M in different concentrations after 5 and 15 minutes of treatment. (B) Densitometric analysis of Western blots in A. The bar plots show band density ratio of pERK/ERK relativized to the negative control,
n = 1. (C) Western blots of total ERK and pERK in Hep G2 cells treated with FGF21-WT, FGF21-Q136V, and FGF21-D117N in different concentrations after 5 and 15 minutes of treatment. (D) Densitometric analysis of Western blots in C. The bar plots show band density ratio of pERK/ERK relativized to the negative control, n = 1. In the experiments, FGF1 at concentration of 1000 ng/ml was used as a positive control; untreated cells were used as a negative control; PCNA and α-tubulin (Tu) were used as a loading control.


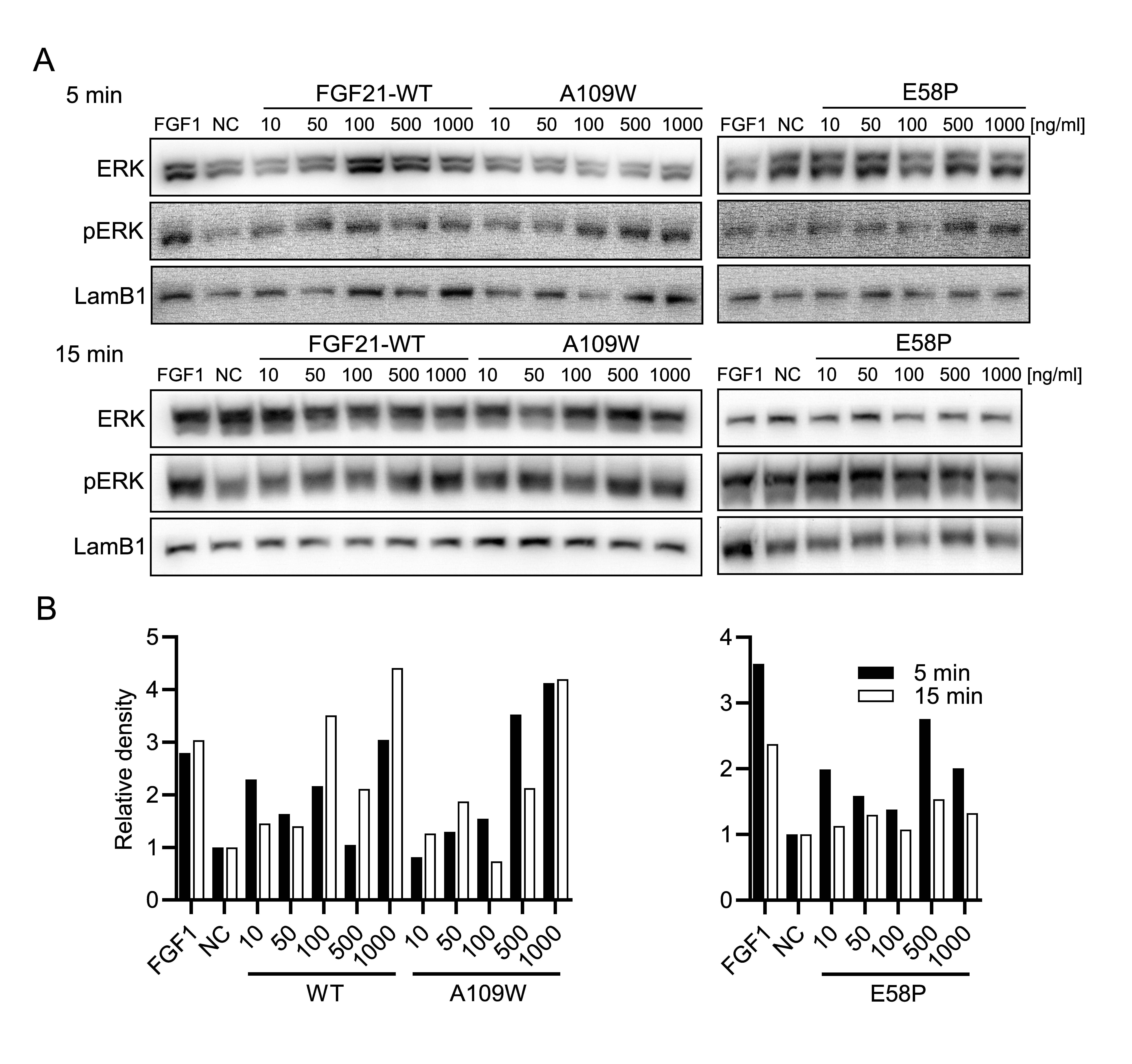


Figure S4. Determination of biological activity of FGF21 variants by activation of ERK pathway. (A) Western blots of total ERK and phosphorylated ERK (pERK) in Hep G2 cells treated with FGF21-WT, FGF21-A109W, and FGF21-E58P in different concentrations after 5 and 15 minutes of treatment. FGF1 at concentration of 1000 ng/ml was used as a positive control; untreated cells were used as a negative control; Lamin B1 (LamB1) was used as a loading control. (B) Densitometric analysis of Western blots. The bar plots show band density ratio of pERK/ERK relativized to the negative control, n = 1.


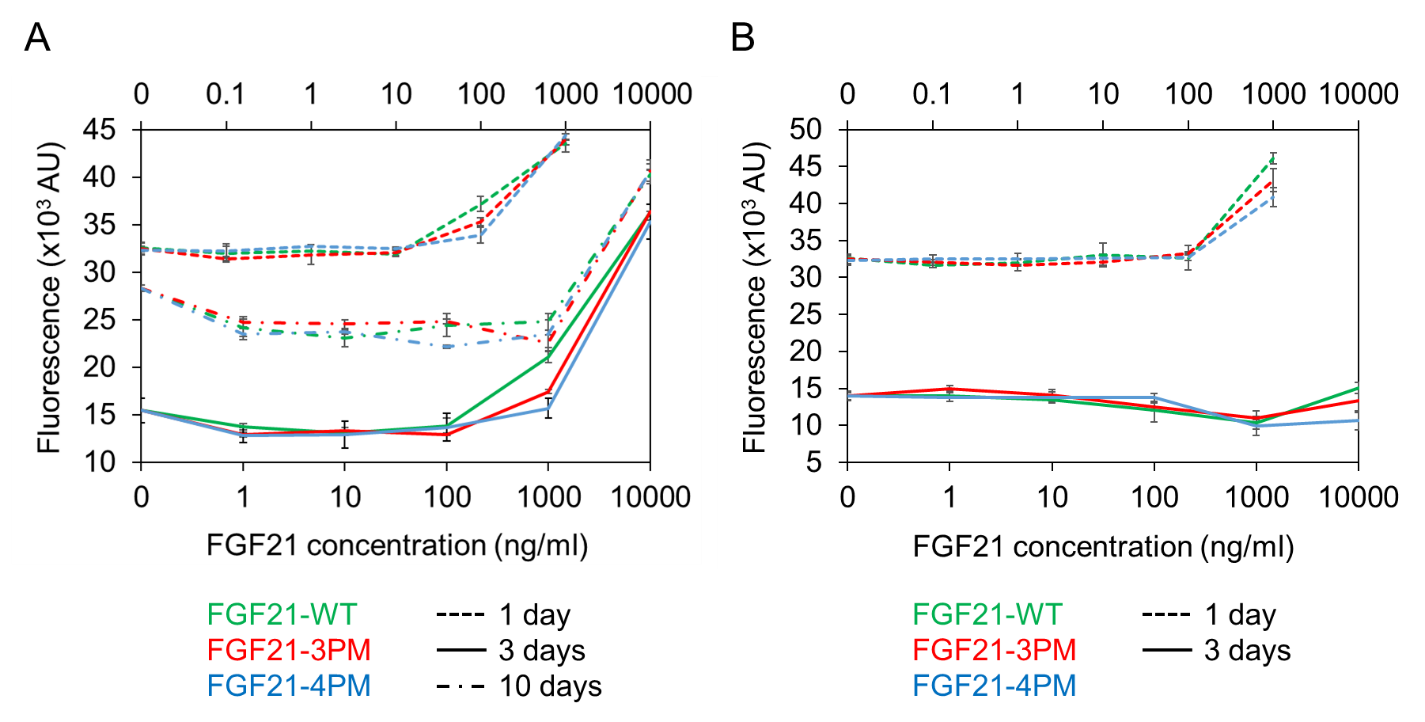


Figure S5. Thermostability testing of FGF21 variants using proliferation assay of Hep G2 cells. FGF21 variants were exposed to (A) 37°C for 1-10 days and (B) 50°C for 1-3 days and then used to treat the Hep G2 cells. The plots show resorufin fluorescence, measured after 4 days of cultivation with FGF21 samples.

References

[1] Zhu L, Zhao H, Liu J, Cai H, Wu B, Liu Z, et al. Dynamic folding modulation generates FGF21 variant against diabetes. EMBO Rep 2021;22:e51352. https://doi.org/10.15252/embr.202051352.

[2] Goetz R, Beenken A, Ibrahimi OA, Kalinina J, Olsen SK, Eliseenkova AV, et al. Molecular insights into the klotho-dependent, endocrine mode of action of fibroblast growth factor 19 subfamily members. Mol Cell Biol 2007;27:3417–28. https://doi.org/10.1128/MCB.02249-06.
